# Three-month pattern of road traffic injuries at a Kenyan level 4 hospital

**DOI:** 10.11604/pamj.2015.20.78.5206

**Published:** 2015-01-29

**Authors:** Duncan Mwangangi Matheka, Mercy Nzilani Kitonyi, Faraj Omar Alkizim

**Affiliations:** 1 School of Medicine, University of Nairobi, Nairobi, Kenya; 2 Young Professionals Chronic Disease Network, Nairobi, Kenya; 3 Centres for Health and Education Programmes, Nairobi, Kenya

**Keywords:** Road traffic crashes, Naivasha, Kenya, vulnerable road users

## Abstract

Road traffic injuries continue to exert a huge burden on the health care system in Kenya. Few studies on the pattern of road traffic injuries have been conducted in Kenya. We therefore carried out a retrospective study to determine the pattern of road traffic injuries seen in a public hospital in Naivasha district, Kenya. A retrospective study on surgical patients admitted at Naivasha District Hospital over a three month period was carried out. Eighty two percent of all patients injured in the crashes were men, and eighty percent were aged between 20-49 years. Most of the patients sustained lower limb injuries (41.5%) followed by head injuries (35.4%). Thirty one percent were pedestrians, 27.7% cyclists, 24.6% passengers and 16.9% motor vehicle drivers at the time of injury. Road traffic crashes are a growing pandemic, leading to high morbidity and mortality. Efforts need to be taken to prevent injuries, long term complications and loss of lives that are robbing communities off loved ones, bread winners and productive manpower.

## Introduction

Road traffic injuries (RTIs) are a leading cause of death and disability worldwide [1]. They account for 1.3 million annual deaths and 20-50 million injuries worldwide [1]. According to the World Health Organization, 90% of these deaths occur in low and middle-income countries (LMICs), which possess less than 50% of global motor vehicles. The African region has the highest road fatality rates in the world's regions (24.1 deaths per 100,000 population), well above the global average of 18.0 deaths per 100,000, in spite of the fact that the region is the least motorized (2% of the world's vehicles) of the six world regions [2]. As such countries continue to develop and expand their road networks, RTIs are expected to rise with the increased usage of roads, increased number of vehicles and increased speed of travel.

Kenya, a low income country in East Africa, has experienced a sharp rise in the number of motor vehicles nationwide. Between the years 2006 and 2010, the total number of newly registered motor vehicles rose almost fourfold, from 52,817 units to 196,456 units per year, with motorcycles accounting for close to 60%. Furthermore there has been an even bigger rise in the number of bicycles, which are being used as taxis, locally known as “boda-boda”. Consequently, Kenya has an estimated death rate of 20.9 per 100,000 population (WHO 2013), which is quite high in comparison to that of the European region (10.3 per 100,000 population) [2, 3]. More than 3000 annual deaths and 60% of admissions to surgical wards in Kenya occur due to RTIs [4]. Such high mortality rates, high morbidity incidences, slow recovery and high incidence of permanent sequelae account for very high medical costs and loss of productivity and gross domestic product (GDP). With the sharp rise in number of vehicles and bicycles on Kenyan roads, this incidence is expected to continue rising. Naivasha district hospital (NDH), a busy level-4 health facility in Kenya has a very high rate of RTI admissions, due to its close proximity to the busy Nairobi-Nakuru highway a major road in the country. The current study therefore aims to document the pattern of RTI admissions seen at the hospital.

## Methods

A retrospective study design was employed to review records of patients admitted in the surgical wards including men and women of all ages seen at the hospital for a three-month period (September-November 2012). Data were collected using researcher-filled data questionnaires that incorporated socio-demographic pattern of the patients involved in the crashes, nature of injury and severity, circumstances that led to the injuries and modes of transport. Data were analyzed using Statistical Package for Social Sciences (SPSS) version 16.0 (SPSS Inc, Chicago, Illinois) and Microsoft Excel and expressed as Mean± SEM. Ethical approval was obtained from the hospital administration and Partnership in innovative medical education Kenya (PRIME-K). All information obtained was confidential and used for the sole purpose of the study.

## Results

***Socio-demographic pattern of the patients:*** Eighty two percent (81.5%) of all patients injured in the crashes were men while 18.5% were women. The age range of patients seen was 3-75 years and the median was 32 years. Eighty percent (80.1%) of the affected people were between 20-49 years (Table 1).

**Table 1:** Age distribution of patients

Age group	No. of patients	Percentage (%)
0-9	6	9.2
10-19	2	3.1
20-29	17	26.2
30-39	23	35.4
40-49	12	18.5
50-59	3	4.6
60-69	1	1.5
70-79	1	1.5
Total	65	100

**Figure 1:**
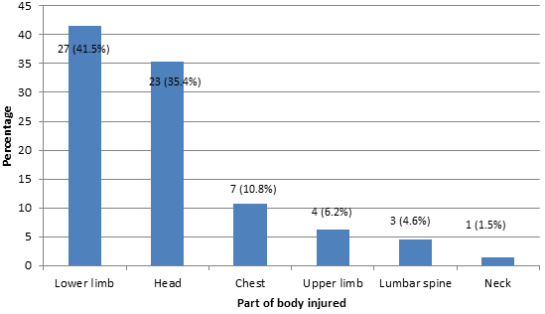
Types of injuries in road traffic crashes

***Nature of injuries:*** Fourty two percent (41.5%) of patients sustained lower limb injuries, 35.4% sustained head injuries, 10.8% sustained chest injuries, 6.2% sustained upper limb injuries, 4.6% sustained lumber spine injuries and 1.5% sustained neck injuries (Figure 1).

**Figure 2:**
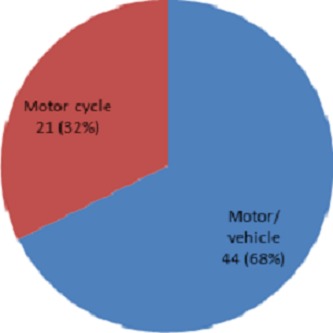
Mode of transport

***Modes of transport:*** Sixty eight percent (68%) of the road traffic injuries involved motor vehicles while 32% involved motorcycles (Figure 2).

**Figure 3:**
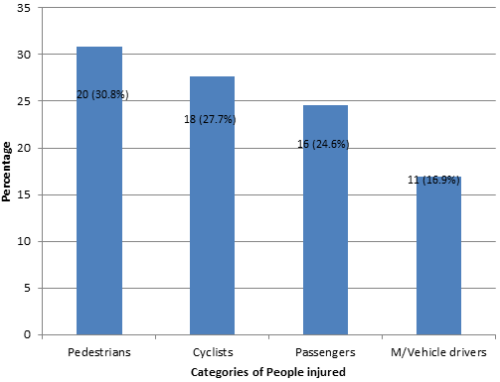
Categories of people involved in the crashes

***Categories of people involved in the crashes:*** Thirty one percent (30.8%) of patients seen were pedestrians, 27.7% were cyclists, 24.6% were passengers and 16.9% were motor vehicle drivers (Figure 3).

## Discussion

Road traffic injuries continue to rise and become a major contributor of morbidity and mortality worldwide [1]. The current study demonstrates that RTIs and deaths in Naivasha mostly affect men between the economically productive ages of 20 to 49 years. Such men are the breadwinners of households, and such injuries, long term sequelae and fatalities therefore have a large scale bearing on communities - socially and economically. On national level, this leads to major losses in productivity and workforce causing serious loss in GDP and economic sustainability. Furthermore, the study also demonstrates that the most affected road users in the area are pedestrians who mostly are purely innocent victims of poor roads, speeding and carelessness of drivers. This may be due to the fact that many roads in Kenya do not have designated pedestrian walk paths and pedestrians are forced to walk on roadsides. Furthermore, lack of designated crossing areas and footbridges, exposes them to danger as they cross roads. Recent surveys have also shown that pedestrians do not utilize footbridges in areas where they are available and prefer to cross roads exposing themselves to the danger of being run-over by speeding vehicles. It is therefore imperative to provide pedestrians suitable footpaths, safe crossing facilities, and to educate them on ways to proactively keep themselves safe from speeding vehicles.

Closely following pedestrians are cyclists who accounted for 28% of victims. They mainly sustained injuries to their heads and limbs, injuries that can be prevented by wearing protective gear such as helmets, limb paddings and reflective jackets. Helmets are made of a shell, impact absorbing liner, comfort padding, face guard and chin strap and are very effective in protecting riders from head injury. Limb paddings effectively protect limbs, while reflective jackets on the other hand increase the visibility of the rider to other road users. Although it is a requirement by law for motorcyclists to put on helmets and reflective jackets, poor sensitization and law enforcement has consistently allowed poor adherence especially in rural areas. Protective gear among bicycle riders on the other hand is nearly unheard of in most parts of the country, exposing them to severe injuries during the unfortunate event of a crash. It is estimated that by the year 2020, road fatalities will increase by 80% in LMICs and decrease by 30% in high-income countries (HICs). Such statistics and those demonstrated by studies such as the current one make it imperative for interventional efforts. Such morbidities and mortalities are easier prevented than most disease associated morbidities and mortalities, and should therefore be given the highest priority in public health and in preventive efforts. A similar study conducted at Kenyatta National Hospital, the highest level of referral and tertiary hospital in Kenya, demonstrated similar patterns with the most common injury due to RTIs being that of lower limb fractures accounting for 27%, followed by head injuries accounting for 25% of RTI admissions. The study revealed that careless driving and negligence by pedestrians are the principal causes of RTIs [5]. The probability that a pedestrian will be killed if hit by a motor vehicle increases dramatically with speed. While most vulnerable road users survive if hit by a car travelling at 30km/hr (90% chance of survival), the majority are killed if hit by a car travelling at 50 km/h (20% chance of survival). Speed regulation is therefore essential in reducing risk of mortality due to road crashes [4].

## Conclusion

Road traffic injuries are a growing pandemic, leading to high morbidity and mortality. Drastic measures need to be undertaken to prevent injuries, long term complications and loss of lives that are robbing communities off loved ones, bread winners and productive manpower.
